# CircOMA1 promotes tumour growth and metastasis of bladder cancer by modulating IGF‐IR/MAPK/EMT pathway

**DOI:** 10.1002/ctm2.983

**Published:** 2022-08-21

**Authors:** Lianghao Zhang, Yonghao Zhan, Longqing Li, Haotian Deng, Jiange Wang, Zhaowei Zhu, Xuepei Zhang

**Affiliations:** ^1^ Department of Urology The First Affiliated Hospital of Zhengzhou University Zhengzhou Henan P. R. China; ^2^ Department of Orthopedic Surgery, West China Hospital Sichuan University Chengdu Sichuan P. R. China; ^3^ Department of Urology Suizhou Central Hospital Suizhou Hubei P. R. China

Dear Editor,

1

Although the prognostic results of patients with early‐stage bladder cancer (BC) are very high, the 5‐year survival rates of patients in advanced stages are greatly reduced to less than 6%.^1^, ^2^ Thus, effective prognostic and therapeutic target biomarkers for early BC diagnostics are urgently needed. Unlike traditional linear RNA, circular RNA (circRNA), because of its covalently closed ring structure and excellent stability even exceeding GAPDH, has a potential application value as a clinical diagnosis and prognostic marker.^3^, ^4^, ^5^ Herein, we revealed the prognostic and diagnostic values of circOMA1 (hsa_circ_100242) and the critical role in promoting progression of BC by modulating insulin‐like growth factor 1 receptor (IGF‐IR)/mitogen‐activated protein kinase (MAPK)/epithelial mesenchymal transition (EMT) pathway. Altogether, our findings offer valuable and novel insights into the mechanism underlying BC regulation and new strategies for clinical practice.

We analysed circRNA expression using CircRNA Microarray technology and identified 382 upregulated circRNAs between 6 pairs of BC tissues and corresponding adjacent noncancerous tissues^6^ (FC > 1.5, *p* < .05, Figure 1A). Our previous study indicated that miR‐145‐5p could inhibit tumour progression through regression of oncogenic IGF‐IR in BC.^7^ Therefore, based on the prediction of Starbase, five upregulated circRNAs that can work as miR‐145‐5p molecular sponges have been screened (Figure 1B). Hsa_circRNA_100242 displayed the highest expression correlation with miR‐145‐5p and was selected for further study (Figure 1C). Hsa_circRNA_100242 is spliced from OMA1 exon two to exon eight and is referred to as circOMA1 (Figure 1D). To confirm the ring structure, we designed convergent and divergent primers and determined that circOMA1 exists as cDNA rather than gDNA (Figure 1E). RNase R digestion assay presented that linear OMA1 was degraded after RNase R treatment, whereas circOMA1 was resistant to RNase R (Figure 1F). Then we confirmed that circOMA1 was upregulated in BC tissue by fluorescence in situ hybridization (FISH) and qRT‐PCR (Figure 1G,H). Based on the median value of circOMA1 expression in BC tissues for patients whose survival data were available, we categorized these patients into the high‐ (*n* = 36) and low‐expression group (*n* = 35). The Kaplan–Meier plot showed that high circOMA1 expression patients had lower overall survival (*p* = .042) (Figure 1I). Furthermore, we found that the high expression of circOMA1 was highly associated with higher tumour grade (*p* = .001), higher pathological T stage (*p* = .036) and lymph node metastasis (*p* = .035) in BC (Figure 1J). The KEGG enrichment analysis revealed that the IGF‐IR/miR‐145‐5p axis most likely affects the downstream MAPK pathway (Figure 1K).

**FIGURE 1:**
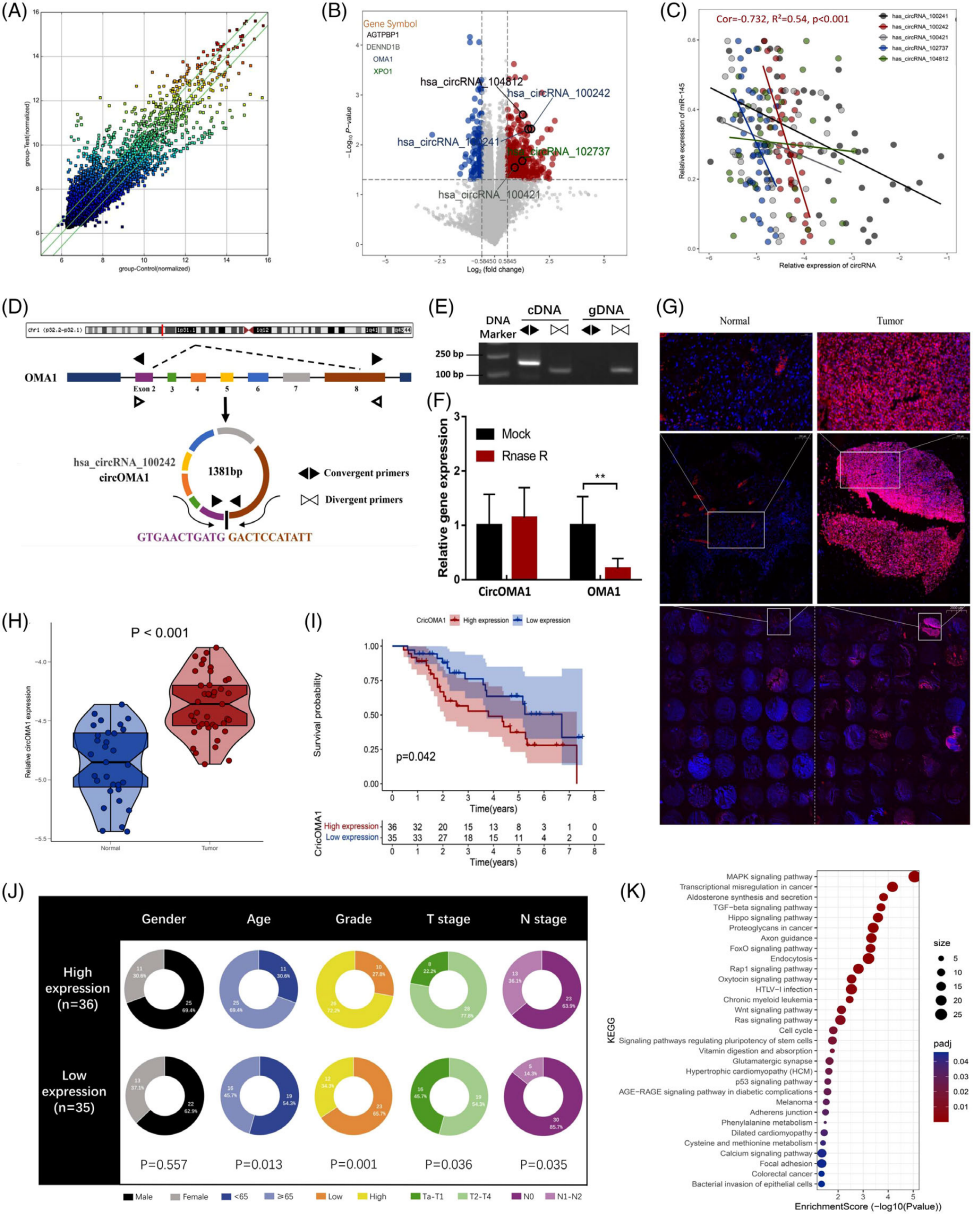
High expression of circOMA1 was identified in bladder cancer (BC) and was related to poor prognosis. (A) The scatter plot was used to assess the differential expression of circular RNAs (circRNAs) between BC tissues and normal tissues. (B) Five upregulated circRNAs from the BC tissues were able to work as miR‐145‐5p sponges. (C) Has_circRNA_100242 displayed the highest expression correlation with miR‐145‐5p. (D) Schematic illustration showing that has_circRNA_100242 (circOMA1) is formed by head‐to‐tail splicing of OMA1 exons 2–8. (E) Agarose gel analysis of PCR production using circOMA1 divergent primer and convergent primer. (F) The qRT‐PCR showed that the circOMA1 and OMA1 expression after RNase R digestion. (G) Fluorescence in situ hybridization (FISH) for circOMA1 (red) and DAPI (blue) in 47 pairs of BC tissue microarray chip. Moreover, circOMA1 locates in BC cell cytoplasm. (H) The qRT‐PCR showed that the relative expression of circOMA1 was higher in BC tissues comparing to paired normal tissues. (I) The Kaplan–Meier plot showed that high circOMA1 expression patients had lower OS than low expression patients. (J) The high expression of circOMA1 was highly associated with higher tumour grade, higher pathological T stage and lymph node metastasis in BC. (K) KEGG enrichment analysis showed that the mitogen‐activated protein kinase (MAPK) pathways were most affected. The data are shown as the mean ± SD. **p < .01

To investigate the function of circOMA1 in BC cells, T24 and 5637 cells were separately transfected with sh‐circOMA1, sh‐NC, OV (overexpression)‐circOMA1 and OV‐NC. We detected the lentivirus transfection efficiency as high as 70% in BC cells by using qRT‐PCR (Figure S1A). In vitro, the proliferation of BC cells was determined by CCK‐8 and EdU assays, respectively. The results indicated that circOMA1 positively regulated T24 and 5637 cells proliferation (Figures 2A,B andS1B–D). Meanwhile, similar results were found on BC xenograft nude mice (Figures 2C and S1E,F). In animal tumour tissues, lower levels of circOMA1 and Ki67 in sh‐circOMA1 xenograft tumours were detected by FISH and immunohistochemistry staining, respectively (Figures 2D and S1G,H). In vitro assays showed that circOMA1 could positively regulate migration (Figures 2E and S1I,J), invasion (Figures 2F and S1K,L) and angiogenesis in BC (Figures 2F and S1M–P). In vivo imaging, the fluorescence intensities of metastatic lung nodules in mice were weaker in sh‐circOMA1 group. Similarly, HE staining results showed that knock‐down of circOMA1 reduced quantity and size of pulmonary metastases (Figures 2G,H and S1Q,R).

**FIGURE 2:**
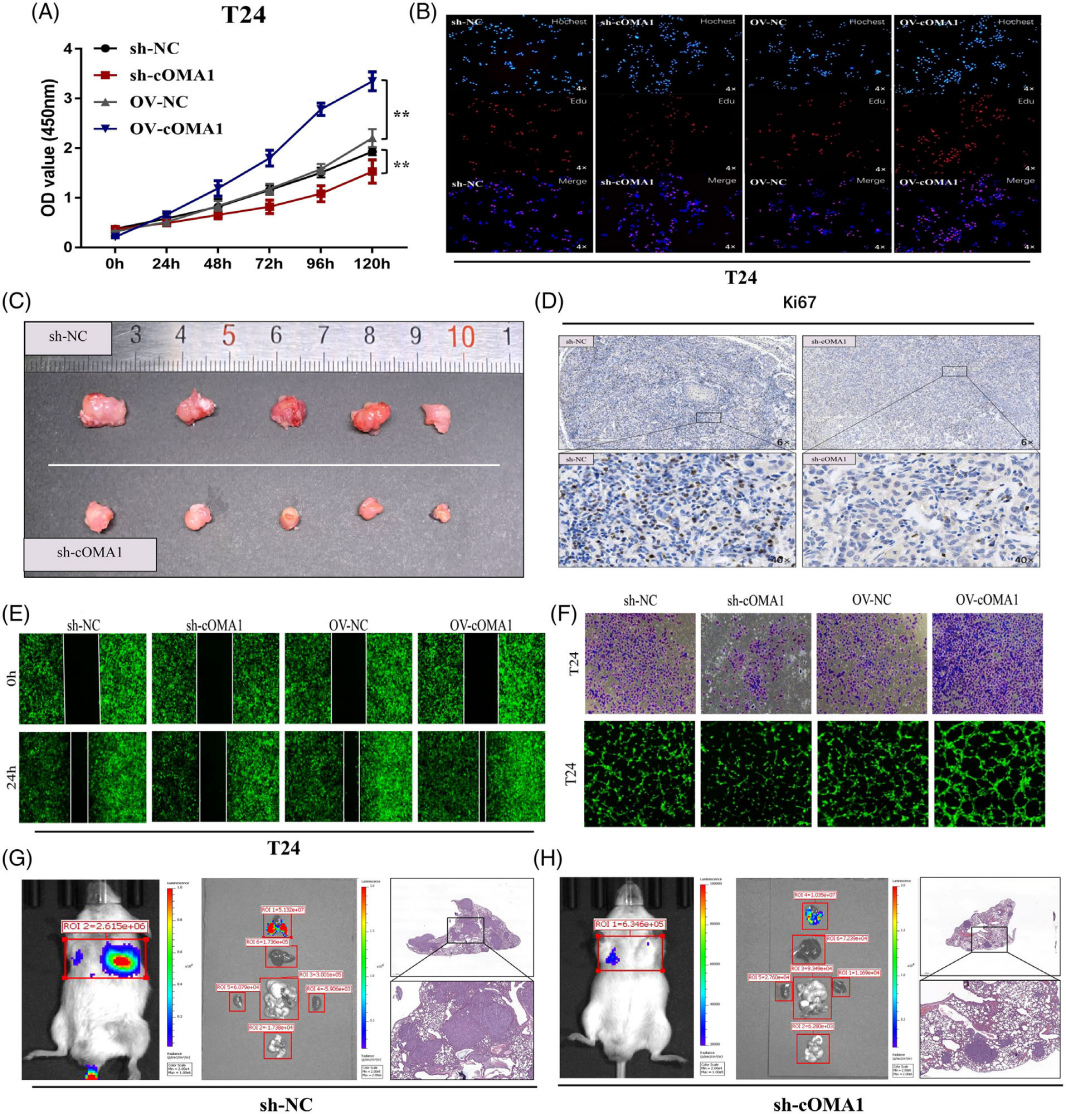
CircOMA1 promotes the proliferation, invasion, migration and angiogenesis of bladder cancer (BC) in vitro and in vivo. (A) CCK‐8 assays were used to evaluate the proliferation changes of BC cells in vitro. (B) EdU assays were used to evaluate the proliferation changes of BC cells in vitro. (C) Tumours collected from nude mice are shown. (D) Immunohistochemistry (IHC) showed the difference of Ki‐67‐positive cells. (E) Wound‐healing assays were used to evaluate the changes of BC cells migratory abilities in vitro. (F) Transwell assays were used to evaluate the changes of BC cells invasion abilities in vitro. HUVEC tube formation assays were used to evaluate the changes of BC cells angiogenesis abilities in vitro. (G and H) The metastasis of BC cells in vivo we also determined using whole‐body fluorescent imaging system, and knock‐down of circOMA1 decreased luciferase signals. The data are shown as the mean ± SD. *p < .05; **p < .01

Double‐labelling immunofluorescence demonstrated that circOMA1 co‐expressed with IGF‐IR (Figure 3A). Moreover, in protein and RNA level, circOMA1 could regulate the expression of IGF‐IR, MAPK family proteins (p‐p38 and p‐p42/44) and EMT (E‐cadherin, N‐cadherin, SNAIL and vimentin) in vitro and in vivo (Figures 3B–D and S2A–C). For the Western blot, the quantifications of protein markers are shown in Figure S2D,E. Based on the previous study,^8^, ^9^ these results indicated that circOMA1 may activate MAPK signalling pathway via regulating IGF‐IR expression to regulate EMT progression. The results of qRT‐PCR revealed that circOMA1 negatively regulated the expression of miR‐145‐5p (Figure S3A). Confocal images showed that circOMA1 was colocalized with miR‐145‐5p prominently in cytoplasm (Figure 3E). Furthermore, higher circOMA1 and miR‐145‐5p levels are found in anti‐AGO2 RIP (Figure 3F). Meanwhile, the results of dual‐luciferase reporter assay showed that miR‐145‐5p decreased the luciferase activity of circOMA1‐WT and IGF‐IR‐WT group, with no effect in circOMA1‐MUT and IGF‐IR‐WT group (Figure 3G,H). Knock‐down of circOMA1 decreased the luciferase activity in IGF‐IR‐WT group (Figure 3I). Afterwards, we also verified the effect of the expression of miR‐145‐5p and IGF‐IR on the prognosis of BC patients and confirmed that the circOMA1/miR‐145‐5p/IGF‐IR regulatory network can affect the prognosis of BC patients (Figure S2F,G). These results indicated that circOMA1 positively regulates IGF‐IR expression via sponging miR‐145‐5p in BC. In the reversal experiment, we silenced miR‐145‐5p in sh‐circOMA1‐transfected cell and overexpressed miR‐145‐5p in OV‐circOMA1‐transfected cell. The IGF‐IR expression of ‘sh‐circOMA1+ inhibitor‐miR‐145‐5p’ group is higher than that of ‘sh‐circOMA1+ inhibitor‐NC’ group, whereas that of ‘OV‐circOMA1+ mimic‐miR‐145‐5p’ group is lower than that of ‘OV‐circOMA1+ mimic‐NC’ group (Figure 4A). Meanwhile, silencing or overexpressing miR‐145‐5p markedly reversed the inhibition or activation of proliferation (Figure 4B–D), invasion (Figure 4E,F) and angiogenesis (Figures 4G,H and S3B,C) in T24 cells. Silencing and upregulation of miR‐145‐5p significantly reversed the RNA and protein levels expression of IGFIR, MAPK and EMT in the sh‐circOMA1 group or OV‐circOMA1 group (Figures 4I,J and S3D). These results indicated that circOMA1 promotes malignant phenotypes of BC cells through positively regulating IGF‐IR expression via miR‐145‐5p‐dependent manner (Figure 4K).

**FIGURE 3:**
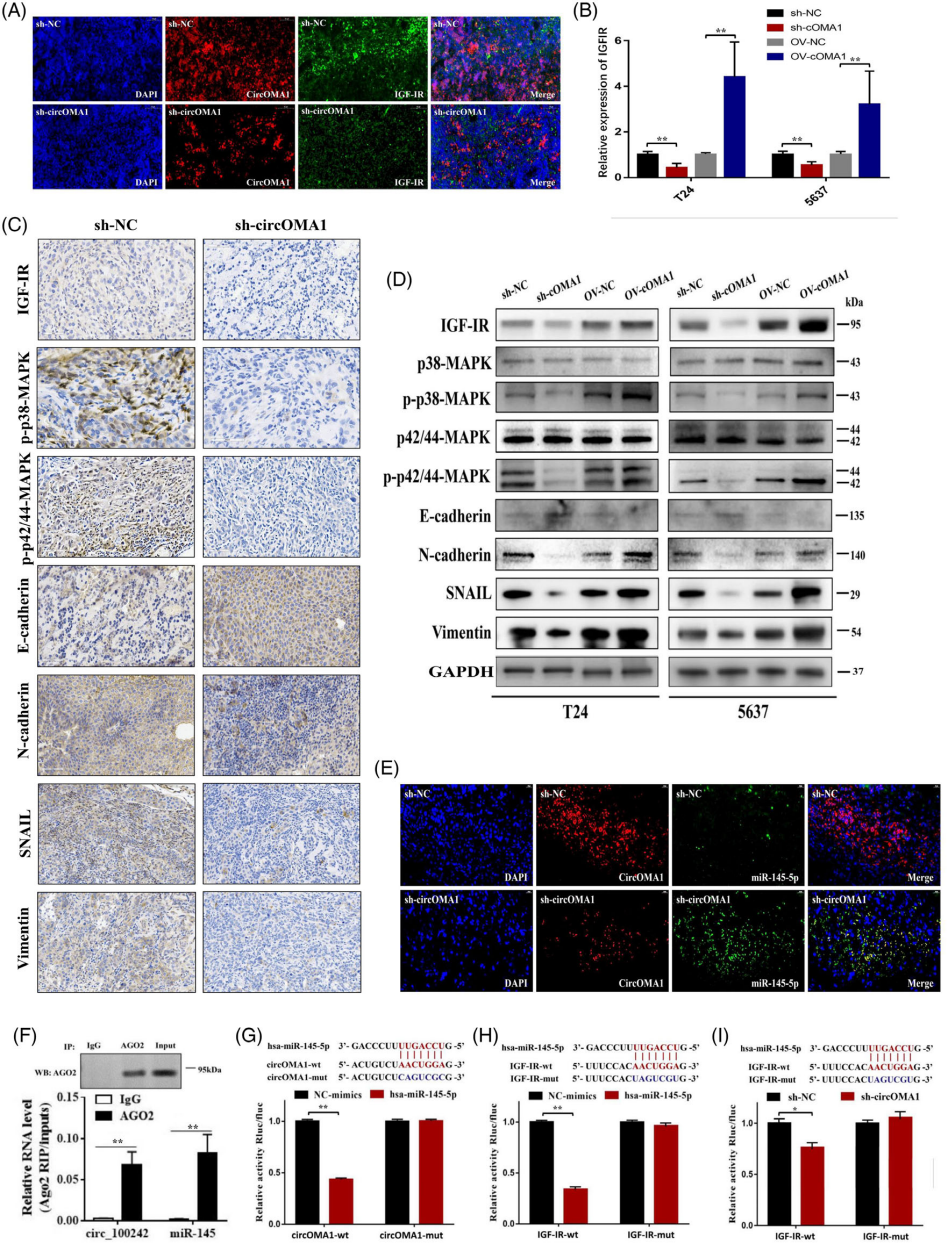
CircOMA1 positively regulates the expression of insulin‐like growth factor 1 receptor (IGF‐IR) via sponging miR‐145‐5p. (A) The expressions of circOMA1 and IGF‐IR were determined by using fluorescence in situ hybridization (FISH), respectively. (B) The results of qRT‐PCR showed that knock‐down or overexpression of circOMA1 markedly reduced or increased the expression of IGF‐IR. (C) The expressions of IGF‐IR/mitogen‐activated protein kinase (MAPK)/epithelial mesenchymal transition (EMT) markers in xenografts were determined using immunohistochemistry (IHC). (D) The Western blotting showed the changes in bladder cancer (BC) cells protein levels of IGF‐IR/MAPK/EMT markers. (E) Co‐localization of circOMA1 (red) and miR‐145‐5p (green). (F) RIP experiments were performed in T24 cells against IgG or AGO2, and the precipitated circOMA1 and miR‐145‐5p were detected by qRT‐PCR. (G) CircOMA1 has putative binding sites with miR‐145‐5p and mimic‐miR‐145‐5p significantly inhibited luciferase activity of circOMA1‐WT group. (H) The 3′UTR sequence of IGF‐IR is complementary to the seed sequence of miR‐145‐5p and mimic‐miR‐145‐5p significantly inhibited luciferase activity of IGF‐IR‐WT group. (I) Knock‐down of circOMA1 decreased the luciferase activity of BC cells transfected with IGF‐IR‐WT. The data are shown as the mean ± SD. *p < .05; **p < .01

**FIGURE 4:**
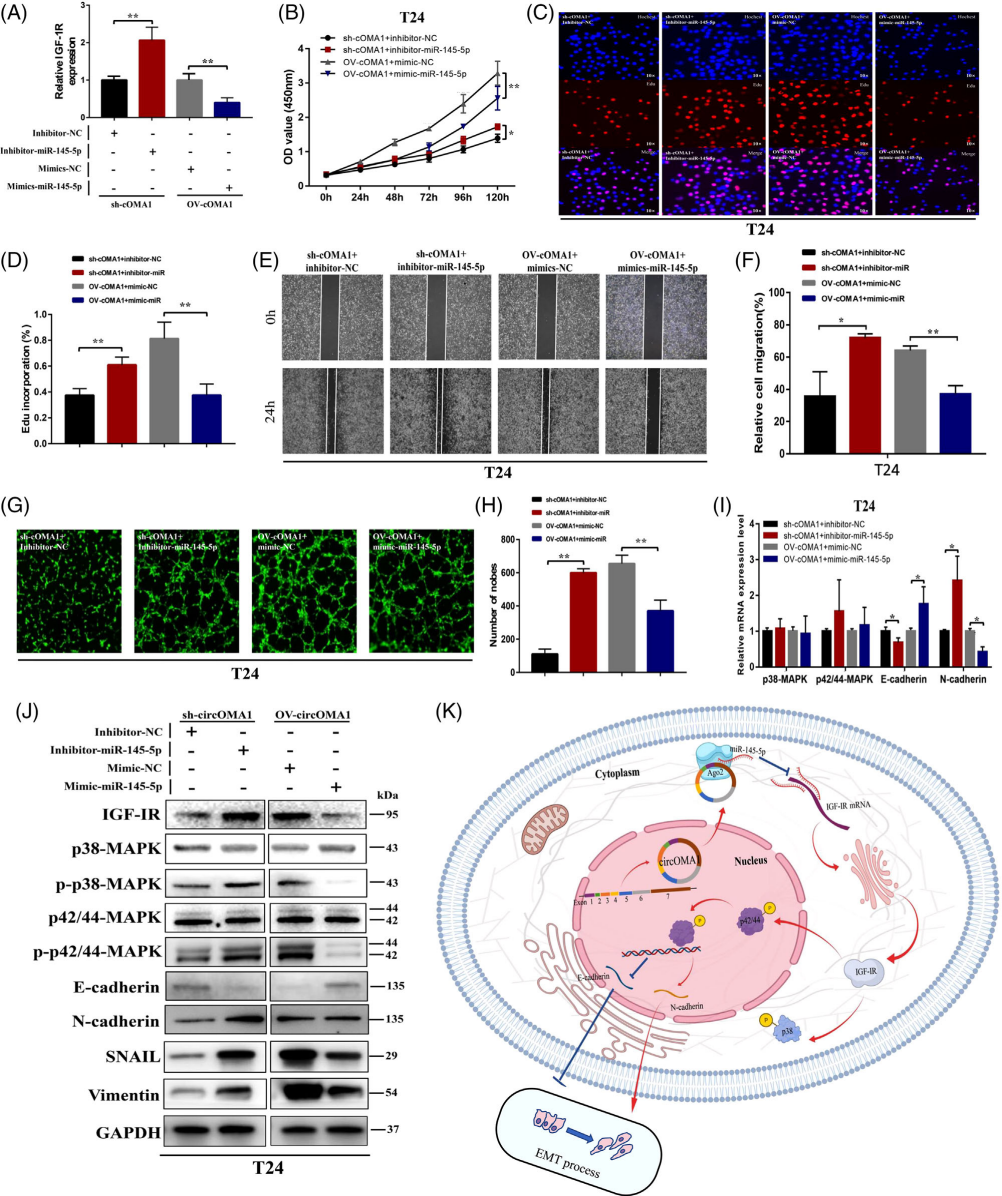
Silencing and overexpressing miR‐145‐5p markedly reverse inhibitory or activation of and proliferation, invasion and angiogenesis in bladder cancer (BC) cell. (A) Knock‐down or overexpression of miR‐145‐5p significantly reversed insulin‐like growth factor 1 receptor (IGF‐IR) expression of T24 transfected with sh‐circOMA1 or OV‐circOMA1. (B–D) Knock‐down or overexpression of miR‐145‐5p significantly reversed the proliferation ability of T24 transfected with sh‐circOMA1 or OV‐circOMA1. (E and F) Knock‐down or overexpression of miR‐145‐5p significantly reversed the invasion ability of T24 transfected with sh‐circOMA1 or OV‐circOMA1. (G and H) Knock‐down or overexpression of miR‐145‐5p significantly reversed the angiogenesis ability of T24 transfected with sh‐circOMA1 or OV‐circOMA1. (I) The qRT‐PCR showed the changes of RNA levels of mitogen‐activated protein kinase (MAPK)/epithelial mesenchymal transition (EMT) markers in T24 cells of reverse experiments. (J) The Western blotting showed the changes of RNA levels of MAPK/EMT markers in T24 cells of reverse experiments. (K) The schematic diagram of the oncogenesis role of circOMA1 in BC. The data are shown as the mean ± SD. *p < .05; **p < .01

In summary, our study is the first to reveal that circOMA1 positively regulates IGF‐IR expression by sponging miR‐145‐5p and activates the MAPK signalling pathway, thus playing a carcinogenic role in the pathogenesis of BC. Furthermore, we propose a ceRNA network, circOMA1/miR‐145‐5p/IGF‐IR, as a potential regulatory mechanism that mediates the occurrence and development of BC. Cumulatively, circOMA1 is a powerful BC regulator and a new therapeutic target for BC treatment that warrants further investigation.^10^

## Supporting information

FIGURE S1 (A) The qRT‐PCR showed that the relative expression of circOMA1 was significantly decreased in knock‐down BC cells and increased in overexpression BC cells. (B) CCK‐8 assays were used to evaluate the proliferation changes of BC cells in vitro. (C‐D) EdU assays were used to evaluate the proliferation changes of BC cells in vitro. (E) Tumor growth of sh‐circOMA1 was slower than that in the sh‐NC group. (F) Tumor weight of sh‐NC group was greater than that in the sh‐circOMA1 group. (G) The expression of circOMA1 was determined by using FISH.(H) IHC showed the difference of Ki‐67‐positive cells. (I–J) Wound healing assays were used to evaluate the changes of BC cells migratory abilities in vitro. (K–L) Transwell assays were used to evaluate the changes of BC cells invasion abilities in vitro. (M–P) Number of nobes formation, number of branches and total branch length in HUVEC tube formation assays were used to evaluate the changes of BC cells angiogenesis abilities. (Q–R) Knockdown of circOMA1 significantly reduced the number of pulmonary metastases and metastases size in vivo. The data are shown as the mean ± SD. ^*^ *P* < 0.05; ^**^ *P* < 0.01.
Figure S2 (A and B) The qRT‐PCR showed the changes in BC cells RNA levels of MAPK/EMT markers. (C) The quantification of IHC expression level (D–E) The quantification of protein markers in each group were detected by western blot. (F) Kaplan–Meier plot showed that high IGF‐IR expression patients had lower OS than low expression patients. (G) Kaplan–Meier plot showed that high miR‐145‐5p expression patients had high OS than low expression patients. The data are shown as the mean ± SD. ^*^ *P* < 0.05; ^**^ *P* < 0.01, ^***^ *P* < 0.001.
Figure S3 (A) The results of qRT‐PCR showed that circOMA1 negatively regulates the expression of miR‐145‐5p in BC cells in vitro. (B–C) Number of branches and total branch length in HUVEC tube formation assays were used to evaluate the changes of BC cells angiogenesis abilities. (D) In rescue experiments, the quantification of protein markers and internal reference proteins in each group were detected by western blot. The data are shown as the mean ± SD. ^*^ *P* < 0.05; ^**^ *P* < 0.01, ^***^ *P* < 0.001.

Supporting Information
