# Supplementary figures and images for: CircOMA1 promotes tumour growth and metastasis of bladder cancer by modulating IGF‐IR/MAPK/EMT pathway
Source: Clin Transl Med. 2022 Aug 21;12(8):e983. doi: 10.1002/ctm2.983 (PMC9393076; doi:10.1002/ctm2.983)

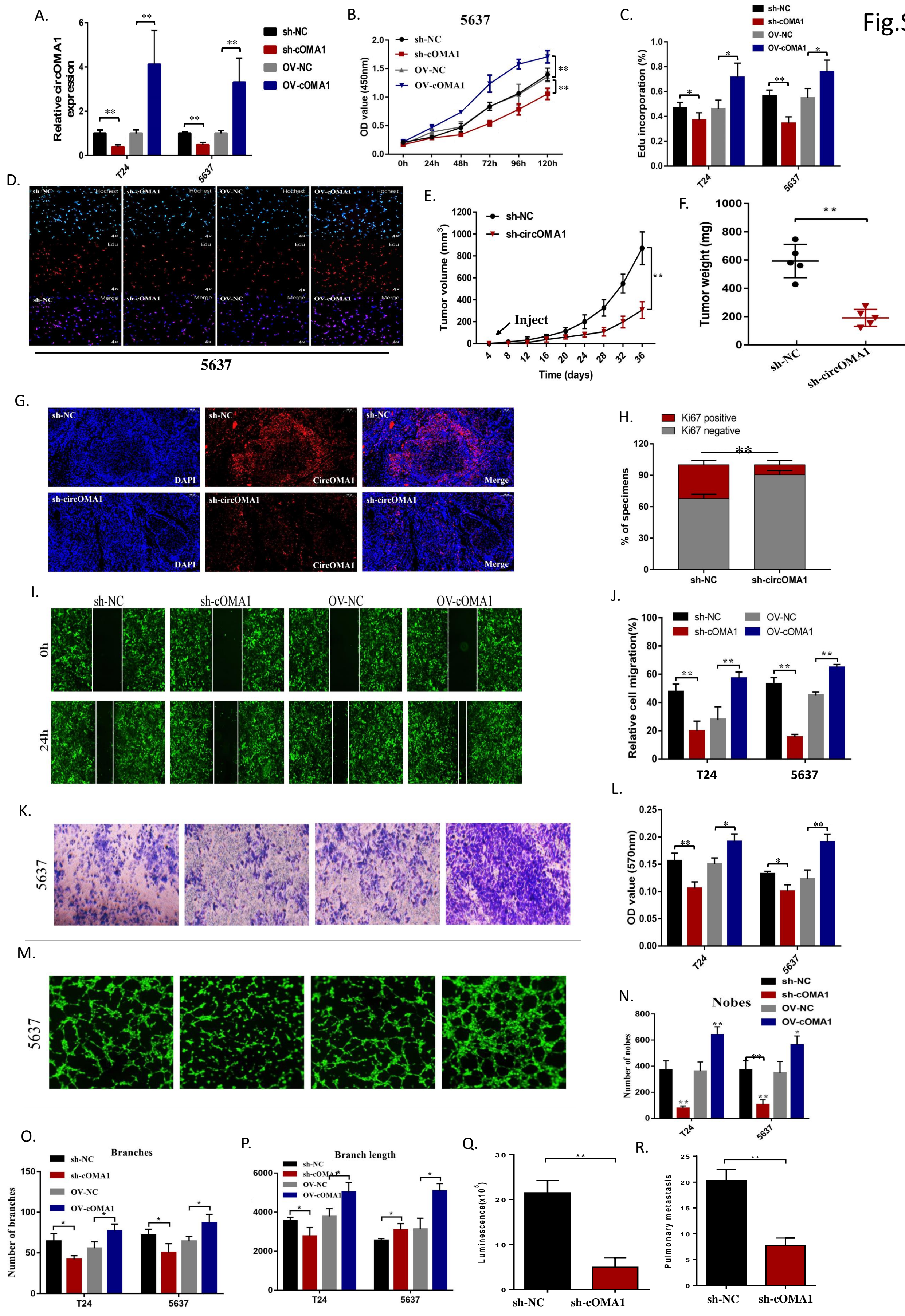

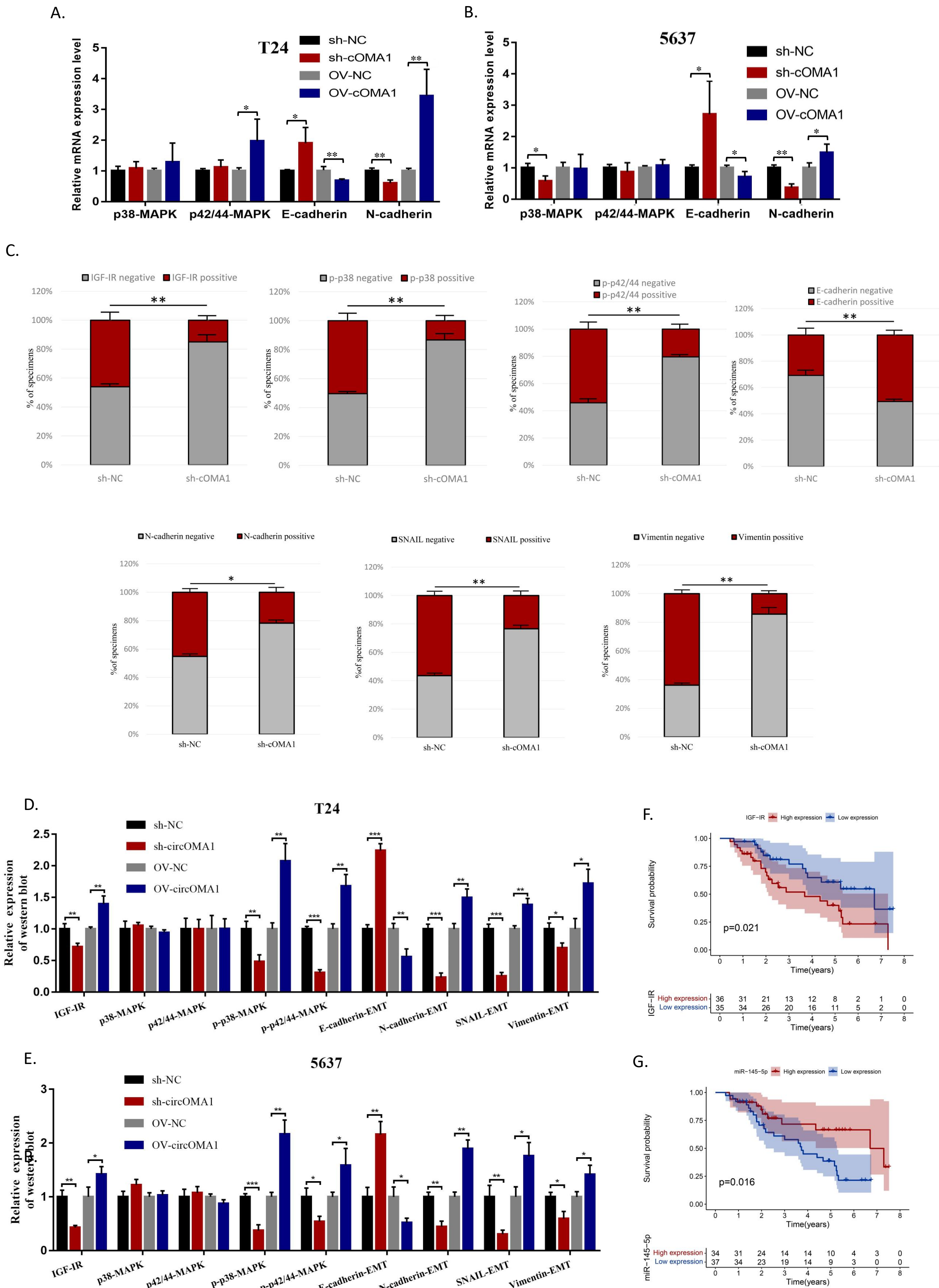

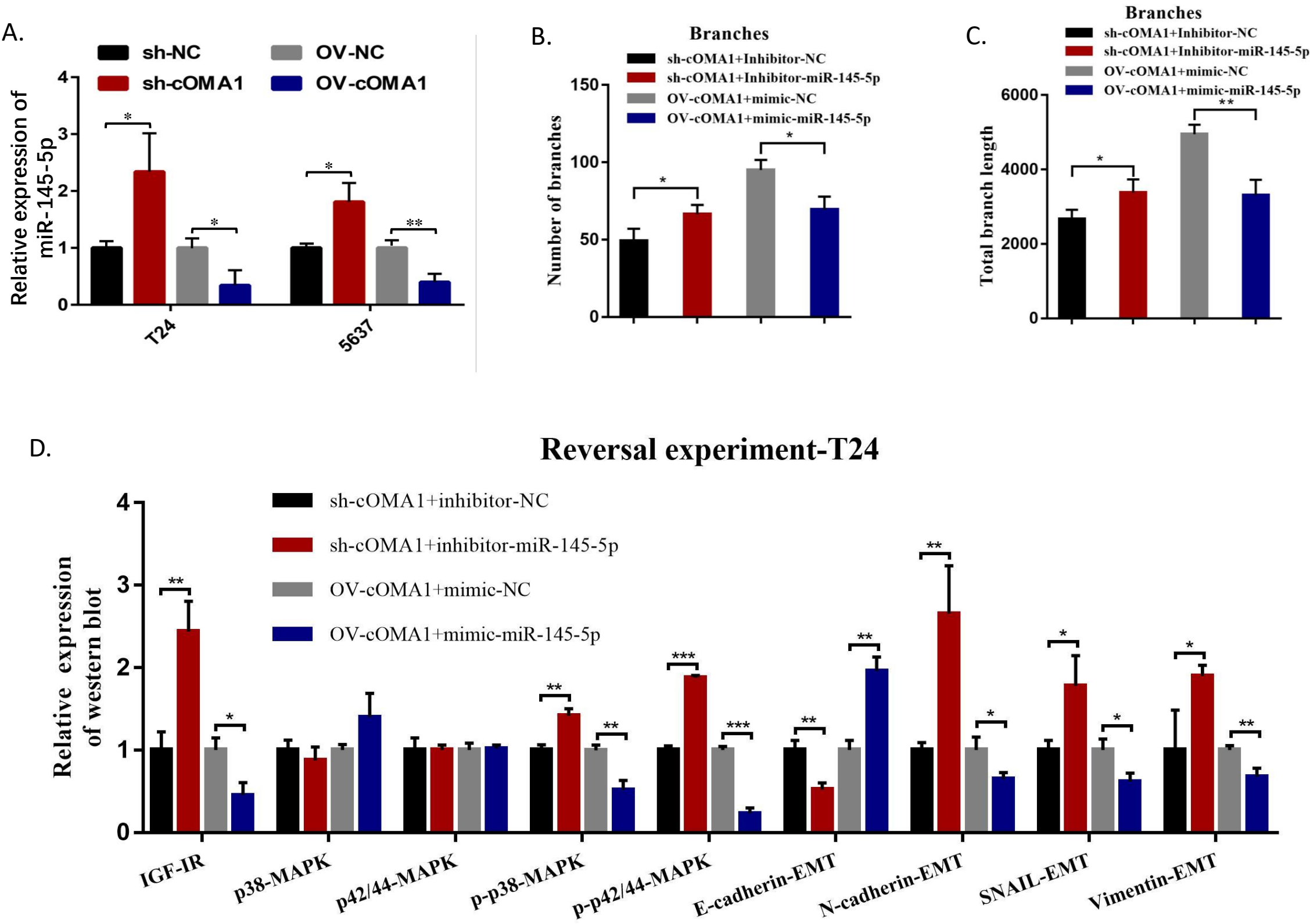

Supplement: Supplementary file 1 — FIGURE S1 (A) The qRT‐PCR showed that the relative expression of circOMA1 was significantly decreased in knock‐down BC cells and increased in overexpression BC cells. (B) CCK‐8 assays were used to evaluate the proliferation changes of BC cells in vitro. (C‐D) EdU assays were used to evaluate the proliferation changes of BC cells in vitro. (E) Tumor growth of sh‐circOMA1 was slower than that in the sh‐NC group. (F) Tumor weight of sh‐NC group was greater than that in the sh‐circOMA1 group. (G) The expression of circOMA1 was determined by using FISH.(H) IHC showed the difference of Ki‐67‐positive cells. (I–J) Wound healing assays were used to evaluate the changes of BC cells migratory abilities in vitro. (K–L) Transwell assays were used to evaluate the changes of BC cells invasion abilities in vitro. (M–P) Number of nobes formation, number of branches and total branch length in HUVEC tube formation assays were used to evaluate the changes of BC cells angiogenesis abilities. (Q–R) Knockdown of circOMA1 significantly reduced the number of pulmonary metastases and metastases size in vivo. The data are shown as the mean ± SD. * P < 0.05; ** P < 0.01. Figure S2 (A and B) The qRT‐PCR showed the changes in BC cells RNA levels of MAPK/EMT markers. (C) The quantification of IHC expression level (D–E) The quantification of protein markers in each group were detected by western blot. (F) Kaplan–Meier plot showed that high IGF‐IR expression patients had lower OS than low expression patients. (G) Kaplan–Meier plot showed that high miR‐145‐5p expression patients had high OS than low expression patients. The data are shown as the mean ± SD. * P < 0.05; ** P < 0.01, *** P < 0.001. Figure S3 (A) The results of qRT‐PCR showed that circOMA1 negatively regulates the expression of miR‐145‐5p in BC cells in vitro. (B–C) Number of branches and total branch length in HUVEC tube formation assays were used to evaluate the changes of BC cells angiogenesis abilities. (D) In rescue experiments, t [file CTM2-12-e983-s001.pdf]
